# Learning-Based Slip Detection for Robotic Fruit Grasping and Manipulation under Leaf Interference

**DOI:** 10.3390/s22155483

**Published:** 2022-07-22

**Authors:** Hongyu Zhou, Jinhui Xiao, Hanwen Kang, Xing Wang, Wesley Au, Chao Chen

**Affiliations:** Laboratory of Motion Generation and Analysis, Faculty of Engineering, Monash University, Clayton, VIC 3800, Australia; hugh.zhou@monash.edu (H.Z.); jxia0020@student.monash.edu (J.X.); hanwen.kang1@monash.edu (H.K.); xing.wang2@monash.edu (X.W.); wesley.au@monash.edu (W.A.)

**Keywords:** slip detection, robotic harvesting, leaf interference, long-short-term memory (LSTM)

## Abstract

Robotic harvesting research has seen significant achievements in the past decade, with breakthroughs being made in machine vision, robot manipulation, autonomous navigation and mapping. However, the missing capability of obstacle handling during the grasping process has severely reduced harvest success rate and limited the overall performance of robotic harvesting. This work focuses on leaf interference caused slip detection and handling, where solutions to robotic grasping in an unstructured environment are proposed. Through analysis of the motion and force of fruit grasping under leaf interference, the connection between object slip caused by leaf interference and inadequate harvest performance is identified for the first time in the literature. A learning-based perception and manipulation method is proposed to detect slip that causes problematic grasps of objects, allowing the robot to implement timely reaction. Our results indicate that the proposed algorithm detects grasp slip with an accuracy of 94%. The proposed sensing-based manipulation demonstrated great potential in robotic fruit harvesting, and could be extended to other pick-place applications.

## 1. Introduction

Robotic harvesting technology has been actively explored for decades as a potential solution to address the increasingly severe labour uncertainty. From the original idea proposed by Schertz and Brown [1] to the fully integrated robotic harvesting systems booming in the past ten years [2,3], significant advancements have been made in machine vision [4,5,6,7], path planning [8], fruit extraction [9], and grasping [10]. However, these achievements in robotic harvesting have yet to secure the level of productivity required to see widespread adoption. In the state-of-the-art, robotic harvesters encounter significant challenges in the presence of unstructured features such as occlusions and obstructions from leaves [11], twigs, and branches [12]. Branches and twigs could affect the accessibility of the target fruit but could be avoided by well-tuned robot path planning and manipulation [13]. On the contrary, obstacles like leaves do not affect accessibility much but can hardly be avoided when performing fruit harvesting tasks. Frequent grasp failures caused by slip have been observed in various robotic harvesting applications [14,15,16]. Experiments conducted by the authors during the 2021 apple harvesting season showed that approximately half of all failed grasps were caused by leaf slippage, making it the leading cause of poor harvesting performance.

In this research, the authors analysed the motion of fruit grasping under leaf interference and identified the reason behind slippage caused grasp failure. A learning-based slip detection and robot manipulation method has been presented to tackle the challenge of leaf interference handling in robotic fruit harvesting. A long-short-term-memory (LSTM) network has been designed for detecting slip between the target fruit and leaf. Experiment results indicated that the proposed LSTM algorithms are capable of detecting leaf slippage, with a 94% accuracy.

The rest of this paper proceeds as follows: Section 2 reviews the related works on fruit grasping and slip detection. Section 3 introduces the methods and materials of the proposed slip detection and robot manipulation. Section 4 presents the experiment setup and related results of the proposed algorithms. Finally, the conclusions are presented in Section 5.

## 2. Literature Review

In the unstructured agricultural environment, fruit harvesting tasks require robotic grasping to be gentle but firm, and capable of handling slip [16]. A substantial amount of research has been carried out to achieve a gentle but firm grasp, while very few investigations have been conducted to enable slip detection and handling feature for harvesting grippers.

Silwal incorporated soft polyurethane pads with an under-actuated end-effector to perform a gentle grasp of apples [17], Bac introduced a gripper with compliant fingers to passively adapt to sweet peppers’ shape [18], and Brown applied a pneumatic soft multi-fingered gripper to conduct flexible plum grasping [19]. Such applications purely rely on the compliance of the fingers and pre-programmed grasping forces without any feedback, thus they can not react to exogenous disturbance like an undesirable slip. Ji [20] and Muscato [21] introduced a grasping force control method with a pair of motor-driven claws for apple picking, and a pneumatic actuated gripper for citrus harvesting, respectively. However, no tactile sensors were integrated into these grippers, resulting in open-loop grasping force control. Tian [22] embedded Interlink FSR-406 force sensors to a two-fingered gripper, and proposed a Fourier transform-based slip detection with an adaptive neuro-fuzzy inference system control method; with such a method, real-time grasping force adjustment was achieved. However, neither the fingers nor the customised sensors are deformable, and the experimental validations are conducted under a highly simplified structured environment, which largely limits its practicality.

In the broader robotic grasping field, slip detection and corresponding robot manipulation have been widely investigated. With steady progress in tactile sensing technology [23], research methods on slip detection can be categorised into four major groups: friction-based methods, vibration-based methods, differentiation-based methods, and learning-based methods [24]. Friction force monitoring is the most intuitive method for slip detection. Many researchers resorted to three-axis tactile sensors to track static friction coefficient [25,26,27]; such a method can achieve slip prediction, but it relies on a multi-axis force sensor which may not be available for many other applications, and its scientific foundation, the Coulomb’s model, is not applicable to soft materials. Vibration-based methods do not require friction information; they have been widely explored by different research teams. Most of the time, researchers [28,29,30] exploit piezoelectric tactile sensors to transform mechanical stimulation into electrical signals, after which various spectral analysis techniques [31,32,33] can be applied to extract essential features for slip, and a number of filters are also utilised to enhance the performance [34,35,36,37]. Such methods are prone to temperature change due to the temperature-sensitive feature of the piezoelectric sensors, but they are still fairly practical if properly designed. Differentiation-based methods require neither friction nor vibration information, and a sharp change of the force derivation can be used to estimate slip [38,39,40]; however, false positives might significantly affect the performance of such methods. With the rapid artificial intelligence development, learning-based methods have attracted increasing attention, Nacy [41] trained an Artificial neural network (ANN) with a back-propagation (B-P) algorithm to optimise grasping force. Van [42] combined spectral analysis with LSTM to achieve slip detection, but it requires two multi-axis force sensors and one bio-mimetic sensor to feed data to the network, which significantly increase the complexity of the system. Veiga [43] adopted random forest regression to classify slip state, but the detection–reaction time is a major pain point.

Above all, despite this valuable progress, existing robotic slip detection approaches need to improve their adaptability on soft robots, and slip detecting and reaction for fruit harvesting have not been achieved in the field.

## 3. Methods and Materials

### 3.1. Slip Analysis

To identify the factors leading to fruit slippage in the robotic harvesting tasks, a slip analysis is conducted as follows. According to the Coulomb friction model, a slip would occur when the tangential force applied by the gripper finger exceeds the max static friction force between the finger and the grasped object. In the case of robotic harvesting, soft fingers are widely adopted, thus the soft-finger contact model [44] needs to be introduced. Compared with the rigid-finger contact model, the soft material allows the contact area to withstand disturbance moments, and the increment of normal force can produce a larger increase of the contact area.

**Figure 1:**
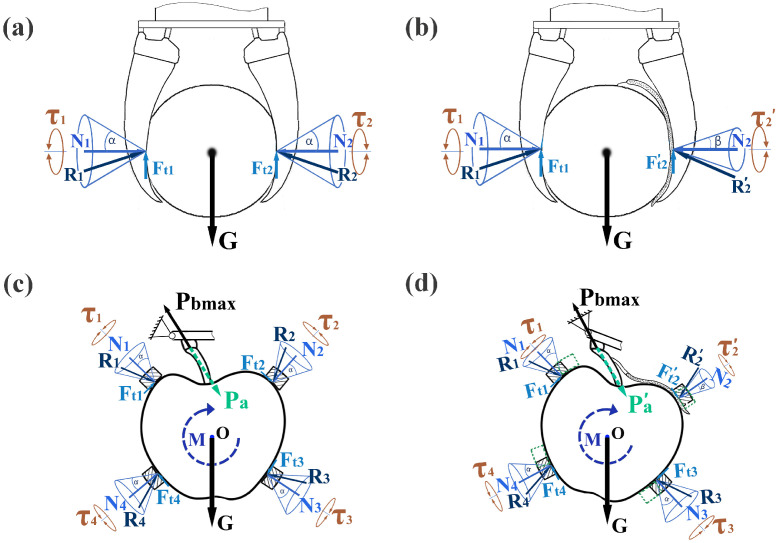
Contact and force analysis of typical grasp scenarios. (**a**) vertical grasp, (**b**) vertical grasp with thin obstacle, (**c**) horizontal grasp, and (**d**) horizontal grasp with leaf interference. Normal forces, equivalent pulling force, and other forces were represented in blue, green, and black color, respectively.

In a typical pick-and-place task, a robot gripper is normally required to lift the object up, as illustrated in Figure 1a. Assuming the multiple contact areas between each finger and the grasped object can be regarded as one equivalent contact area, the equilibrium conditions with contact constrains are:

(1) $$\left\{\begin{array}{c} \sum_{i=1}^{n}\left(F_{i}\right)=G+\sum_{i=1}^{n}\left(R_{i}\right)=0\\ \sum_{i=1}^{n}\tau_{i}=0 \end{array}\right.$$

where *n* is the number of total fingers applied to grasp the object, *G* is the gravity of the target object, $R_{i}$ is the resultant of normal force $N_{i}$ and tangential force $F_{ti}$ applied by the $i^{th}$ finger, $\tau_{i}$ is the moment exerted on the $i^{th}$ finger, and

(2) $$F_{ti}=\mu_{0}N_{i}\leq F_{fm}$$

where $\mu_{0}$ is the friction coefficient between finger surface and the target object, and $F_{fm}$ is the maximum static friction force. To prevent slip, the resultant vector $R_{i}$ must be within the corresponding friction cone. When an undesirable thin obstacle is grasped between one of the fingers and the target object, as shown in Figure 1b, the tangential force will be changed and the direction of the resultant force will be shifted accordingly, if either $\mu_{0}$ or the friction coefficient between the target object and the obstacle or between the obstacle and the finger is smaller than $\mu_{0}$; with the same normal force, the tangential force will be reduced and a slip might occur.

In the fruit harvesting task, the picking motion typically involves a twisting motion [45] to detach the target fruit from the twig and a horizontal motion to transport the picked fruit, if we assume all the fingers are directly in contact with the fruit, as shown in Figure 1c, the twisting moment *M* will be executed by the equivalent tangential forces $F_{t1},F_{t2},F_{t3},F_{t4}$ on the fingers, which will drive the target fruit rotating along the center of the gripper, causing an equivalent pulling force $P_{a}$ in the junction point of fruit stem and the twig. To secure a successful detachment, the equivalent pulling force has to outweigh the maximum pulling force that can be withstood by the twig $P_{bmax}$:

(3) $$P_{a}=\frac{\sum_{i=1}^{n}F_{ti}d_{i}}{d_{a}}\geq P_{bmax}$$

where $d_{i}$ is the distance between the contact area of the *i*th finger and the object center of mass (COM), and $d_{a}$ is the distance between the pulling force axis and the COM.

When there is a piece of leaf grasped by the *j*th finger, the tangential force will be affected by the changed friction coefficient $\mu_{j}$, and the equivalent pulling force becomes:

(4) $${P_{a}}^{\prime }=\frac{\left(\sum_{i=1,i\neq j}^{n}N_{i}\times \mu_{0}\right)d_{i}+\left(N_{j}\times \mu_{j}\right)d_{j}}{d_{a}}$$

From (3) and (4), the change of the equivalent pulling force is:

(5) $$P_{a}-{P_{a}}^{\prime }=\frac{d_{j}}{d_{a}}\left(N_{j}\times \left(\mu_{0}-\mu_{j}\right)\right)$$

Therefore, we have:

(6) $$P_{a}>{P_{a}}^{\prime }\;if\;\mu_{j}<\mu_{0}$$

Therefore, if the friction coefficient of leaf-fruit is smaller than that of finger-fruit, the twisting moment *M* may not be sufficient to secure a successful fruit detaching. The authors conducted tests to compare the two friction coefficients, and found that the friction coefficient of leaf-fruit is 4.3–7.5 times, and 3.0–10.0 times smaller than the silicone-fruit friction coefficient in dry and wet conditions, respectively. Furthermore, the reduced maximum static friction force between the finger and the fruit could cause a slippage on that finger. It is important to note that, when the fingers are made of flexible material, the slippage on one finger can eventually lead to a slip off of the target object. As illustrated in Figure 1d, when the end joint of the robot arm applied a twisting moment to the gripper base, the finger base will rotate to the designated area (the green dotted area), while the finger tip or the contact areas tend to lag behind due to the effect of friction force. However, when a leaf was grasped between *i*th finger (take the 2nd finger as an example) and the target fruit, the relatively smaller friction coefficient could cause a slippage so that the contact area of the 2nd finger tends to rotate smoothly with its finger base, then the distance between the actual position of the two contact area on the 1st finger and the 2nd will be enlarged. If the enlarged distance exceeds the diameter of the target fruit, the dragging force of the branch could pull the fruit off the gripper at a certain point. This explains the slip motion pattern observed in the robotic apple harvesting field trials. Therefore, a method to detect the slip and control the finger-fruit friction force is essential for improving fruit harvesting success rate.

It is worth mentioning that the above analysis is based on the scenario where the leaf interference only occurred on one finger; the scenarios of leaf interference on multiple fingers simultaneously are not included. In fact, the authors analysed the previous field test videos (831 apples grasped) and noticed that the scenario of leaf interference on multiple fingers simultaneously only accounts for 19.1% (159 grasps); therefore, in this work, we take the task of slip detection on one finger as a start.

### 3.2. System Architecture

To enable robots with slip detection and handling features, a combination of essential gripper dexterity, real-time tactile feedback, and closed-loop gripper control is required.

Specifically, essential gripper dexterity requires the fingers to be independently controlled with a certain degree of freedom, and real-time tactile feedback indicates that robust sensing hardware and software need to be integrated into the end-effector system, with which a closed-loop control can be achieved simultaneously.

**Figure 2:**
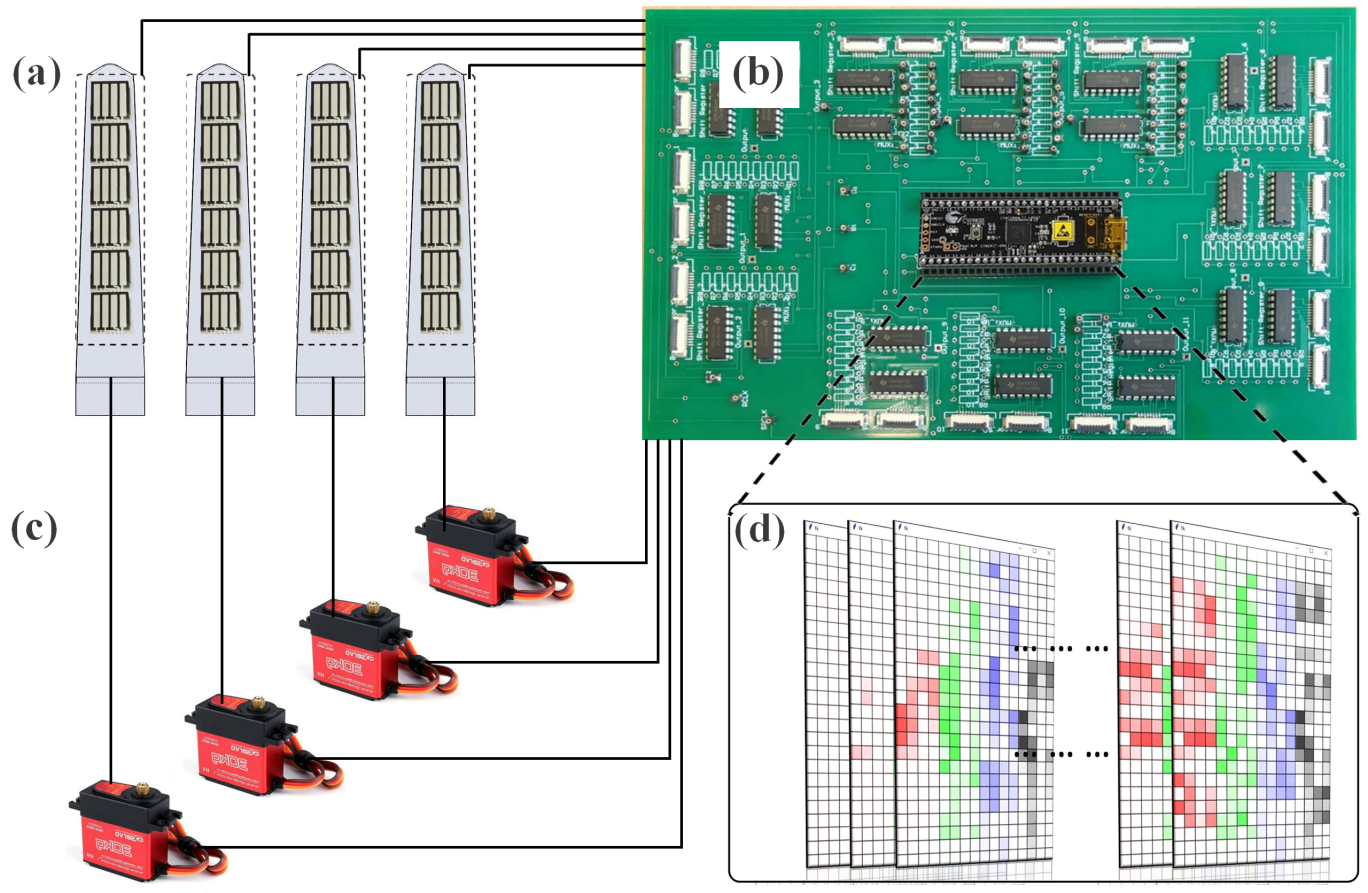
System architecture. (**a**) data collection; (**b**) data processing; (**c**) control and manipulation; (**d**) tactile perception (tactile data collected in four fingers will be represented with red, green, blue and gray colors in the visualisation, larger force applied on the sensing area will generate darker shade of the color).

For these purposes, a system architecture was carried out as shown in Figure 2. Tactile sensors will be embedded into the fingers so that real-time tactile feedback can be collected, after which the data will be processed in a customised Data Processing Unit (DPU), where the data perception will be performed. The DPU will then send control commands to the actuators based on the perception outcome, and the actuators will eventually execute the planned motion of the fingers to handle the slip disturbance.

### 3.3. Gripper Construction

**Figure 3:**
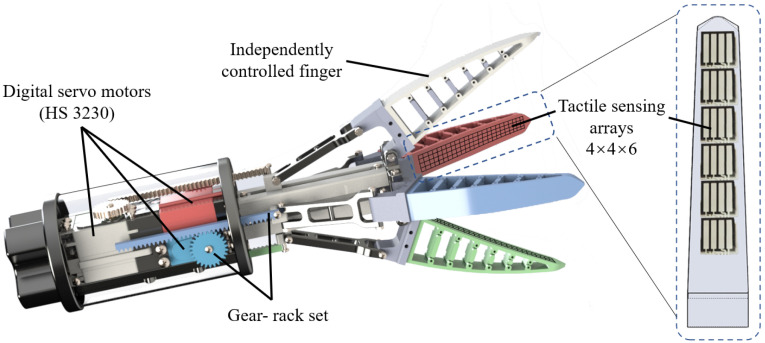
Gripper with independent motor-controlled fingers.

To achieve the desired dexterity proposed in the system architecture, a 4 Degree of Freedom (DoF) under-actuated gripper has been designed and constructed as shown in Figure 3. The four-fingered design of this gripper is to reduce the potential apple bruise by increasing contact area, and to provide essential redundancy under challenging picking scenarios, so that, if one finger is affected by the leaf (or other obstacles) interference, the remaining fingers could still secure a stable grasp and detachment.

The four flexible fingers with fin-ray structures were printed with Thermoplastic Polyurethane (TPU) filaments. A layer of ELASTOSIL RTV3428 silicone was moulded on the finger surface to increase the friction coefficient. The silicone skin can also reduce force concentration to mitigate potential mechanical damage. Each finger is actuated by an independent HS3230 servo motor through a rack-gear set; when the motor rotates clockwise, the gear will drive the rack forward so that the attached compliant finger will be pushed against the grasped object, and the fin-ray structure will deform and adapt to the target fruit’s shape and size. When the motor rotates to the opposite direction, the finger will be pulled back and the force exerted by the finger will be steadily reduced. In this way, the grasping force can be adjusted by controlling the motor rotation angles, and essential dexterity can be provided by adjusting the grasping force independently on four fingers.

### 3.4. Sensor Integration

To provide the gripper with proposed real-time tactile sensing ability, piezoresistive tactile sensors were selected for their excellent power-saving feature, cost efficiency and reliability [46]. Specifically, 24 off-the-shelf RX-M0404S tactile arrays were embedded in the four fingers under the silicone skin. The tactile array sensors were double taped on the finger skeleton along the longitudinal centre line and then soaked inside the liquid silicone mud when moulding, so that potential sensor displacement could be prevented. Each tactile array is 14 mm × 14 mm by size and 4 × 4 by taxel elements. Therefore, there are 96 taxels in the 6 tactile arrays on each finger.

Any external force between 0.2 N and 20 N will trigger a resistance change of the taxel units. The resistance change will be transformed to voltage change via a voltage divider circuit and thus been measured by a customised data processing unit. The customised data processing circuit adopted the concept of signal isolation [47] to minimise the crosstalk among the 384 taxels. We regard each taxel as a variable resistor and ultilise an electrical-grounding-based readout architecture to read the resistor values one after another. Specifically, we feed one side with a constant voltage while connecting the other side of the taxel to a shift register. The shift registers (Texas Instruments-SN74HC595) will selectively provide either the constant voltage or 0 volts (grounded). In such a way, potential crosstalk during the measurement of each taxel’s feedback can be prevented.

The measurements will be sent via twenty four 8-pins Flat Flex Cables (FFC) to the selected channels of multiplexers (Texas Instruments-CD4051BE), and then be stored in a Cypress PSoC5 controller for further processing and perception.

### 3.5. Learning-Based Slip Perception

To decode the tactile data provided by the 384 taxels, deep-learning-based algorithms are to be implemented. Since the tactile information to be processed in this work is high-dimensional spatio-temporal data, Recurrent Neural Networks (RNN) are preferred for their intrinsic capabilities to capture time-dependent behaviors [48]. Convolutional Neural Networks (CNN) that are prevailing in the image processing domain are not sufficiently competent to extract slip features in real-time, as they require the temporal characteristics to be transformed into spatial characteristics before perception, which tends to be time-consuming and energy inefficient [49].

Although RNNs are good at extracting time series features, frequent occurred exploding or vanishing gradients make them very difficult to be trained. Thus, Long Short-Term Memory (LSTM) recurrent networks are to be utilised in this work, as LSTM networks overcome these issues by introducing memory cells via self-parameterized controlling gates to store, access, write, and clear tactile information. Such trait makes LSTM excel at temporal signatures classification and pattern identifying [42], and therefore ideal for slip detection in this work.

### 3.6. Closed-Loop Grasp Manipulation

**Figure 4:**
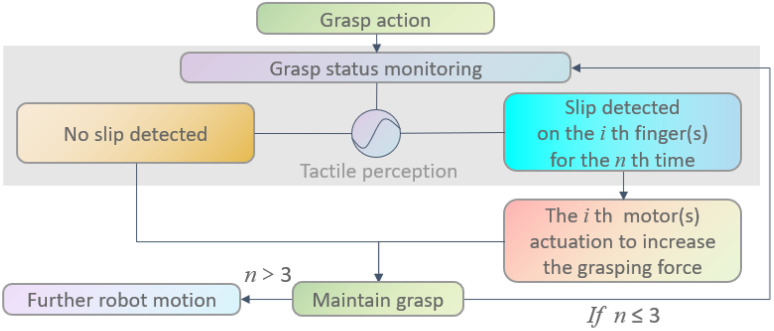
Control flow diagram of the proposed closed-loop grasp manipulation.

The following closed-loop control has been applied in this work to address the grasp manipulation task under leaf interference. As presented in Figure 4, a grasp action will be initialised after receiving a grasp command, and followed by continuous grasp status monitoring, where the proposed tactile slip perception will be performed. If no slip is detected, the gripper will maintain grasp without additional action. If a slip been detected by one or multiple finger(s), the corresponding motor(s) will be actuated to increase the grasping force on the specific finger(s)—after which, the grasp will be maintained for next round slip detection, and further robot motion command will be executed if no slip is detected in this round or if the total times of the grasping force adjustment exceeds 3.

It is important to note that the increased grasping force might cause apple damage. According to Ji et al.’s [50,51] research, the maximum stress that can be withstood by both apple (Fuji) flesh and apple skin before irreversible damage is 0.31 Mpa. To prevent the grasping force from damaging the fruit, the motors of the proposed gripper were tuned to provide contact pressure under 0.31 MPa.

**Figure 5:**
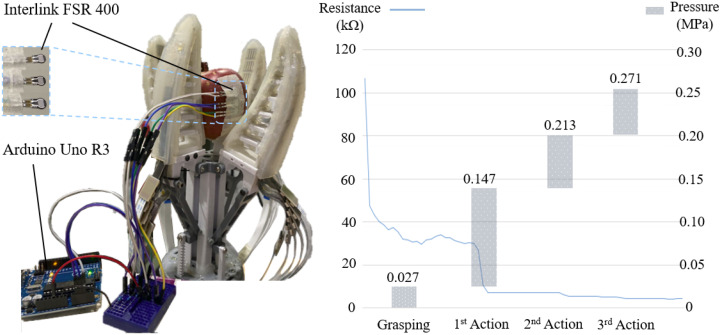
Grasp force tuning of the designed grasp manipulation.

As shown in Figure 5, the designed motor rotation angles were tuned by measuring the contacting force data during the grasp manipulation process. Three off-the-shelf force sensors (Interlink FSR 400) were utilised to output the real-time resistance value, and the maximum resistance value collected by the three sensors was adopted to retrieve the contact force as per the Resistance-Force chart provided by the FSR 400 supplier. The contact force was then transformed to pressure value (by dividing the 20.27 mm${}^{2}$ active sensing area) in the unit of MPa. After tuning, the proposed gripper was able to limit the output finger-fruit pressure under 0.31 MPa (based on 100 grasp measurements).

It should be noted that the 0.31 MPa threshold is set for Fuji apples with specific weight and volume [50]. Apples with different weight, volume, and variety may have lower maximum withstandable stress threshold. Therefore, it is important to keep the contact pressure to a low level (just enough to secure a grasp of dry apple with a diameter between 60 mm and 100 mm) and increase it only when necessary. In this work, the initial contact pressure between the fruit and the finger was tuned to be less than 1/10 of the 0.31 MPa threshold, The right part of Figure 5 presents an example of the tuned contact pressure control during the planned grasp-manipulation process. Specifically, the proposed gripper exerted up to 0.027 MPa, 0.147 MPa, 0.213 MPa, and 0.271 MPa pressure for the initial grasping motion, 1st action motion, 2nd action motion, and 3rd action motion, respectively.

## 4. Experiment and Discussion

Experiments were conducted to validate the proposed solution of learning-based slip detection and grasp manipulation. Since a slip detection result is essential to trigger grasp manipulation, the experiments were focused on the slip detection algorithm validation.

A supervised binary classification method was adopted to detect slip signatures during the grasping process, where sequential tactile data were divided into a specific time window and categorised into two distinct groups: slip and non-slip. To make it easier for slip localisation and performance assessment, the slip group was further breakdown into four subsets: slip on finger 1, slip on finger 2, slip on finger 3 and slip on finger 4.

### 4.1. Neural Network Architecture

**Figure 6:**
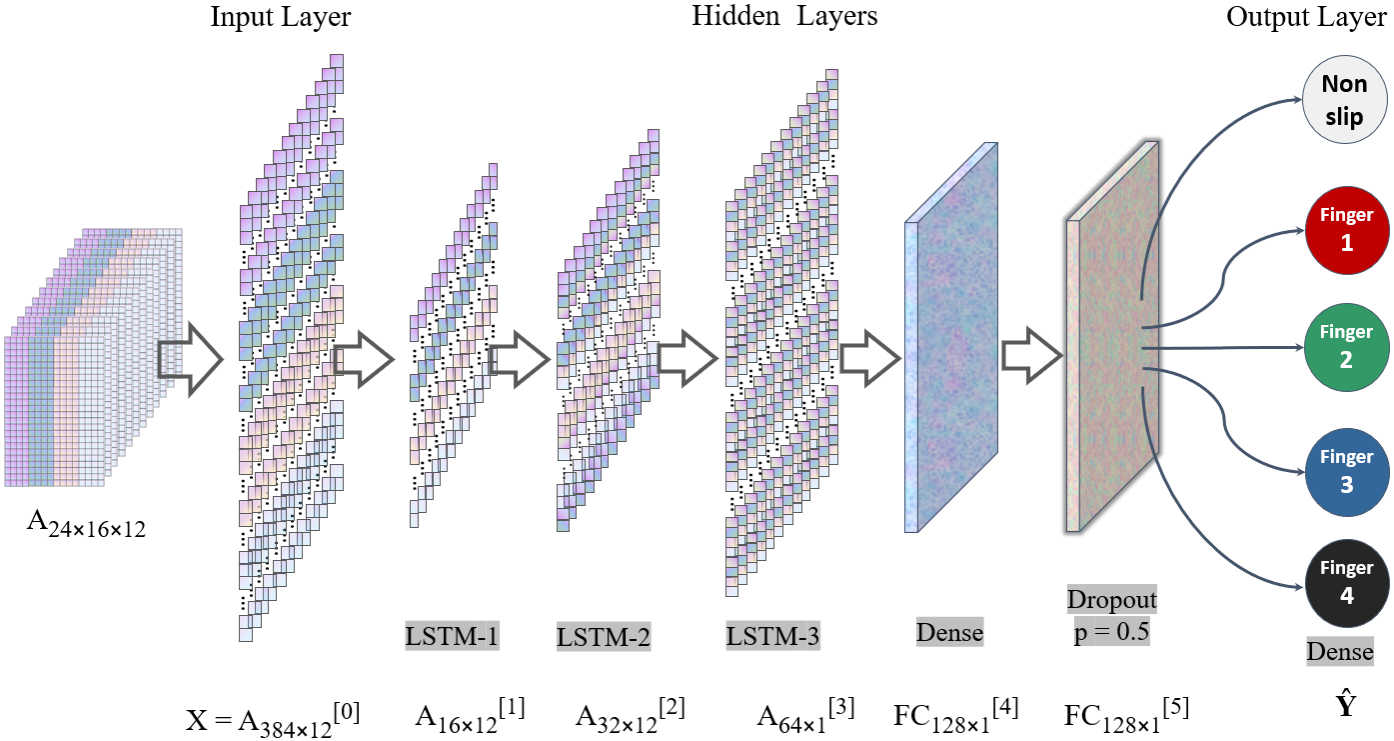
Proposed slip detection neural network architecture.

The proposed LSTM network contains input layer, hidden layers, and output layer, as shown in Figure 6.

The input layer is designed to feed a 384 × 12 matrix to the hidden layers, where three long-short term memory layers with 16, 32, and 64 units are set one after the other. Each LSTM layer will learn and keep a few selected features while discarding certain unimportant information, and the data size will be enlarged from a 16 × 12 matrix in the first LSTM layer to 32 × 12 matrix in the second LSTM layer and then 64 × 12 matrix in the third LSTM layer. The three-layers combination is designed to enhance the learning ability of the network and improve the convergence time. Two fully connected layers are placed after the three LSTM layers to dense and dropout the processed information—after which, the second fully connected layer is designed to output the classification outcome as five groups. We ultilise a SoftMax [52] with K = 5 as the activation function, where one-hot method [53] has been applied to produce a faster clock rate running of a state machine.

### 4.2. Data Collection

**Figure 7:**
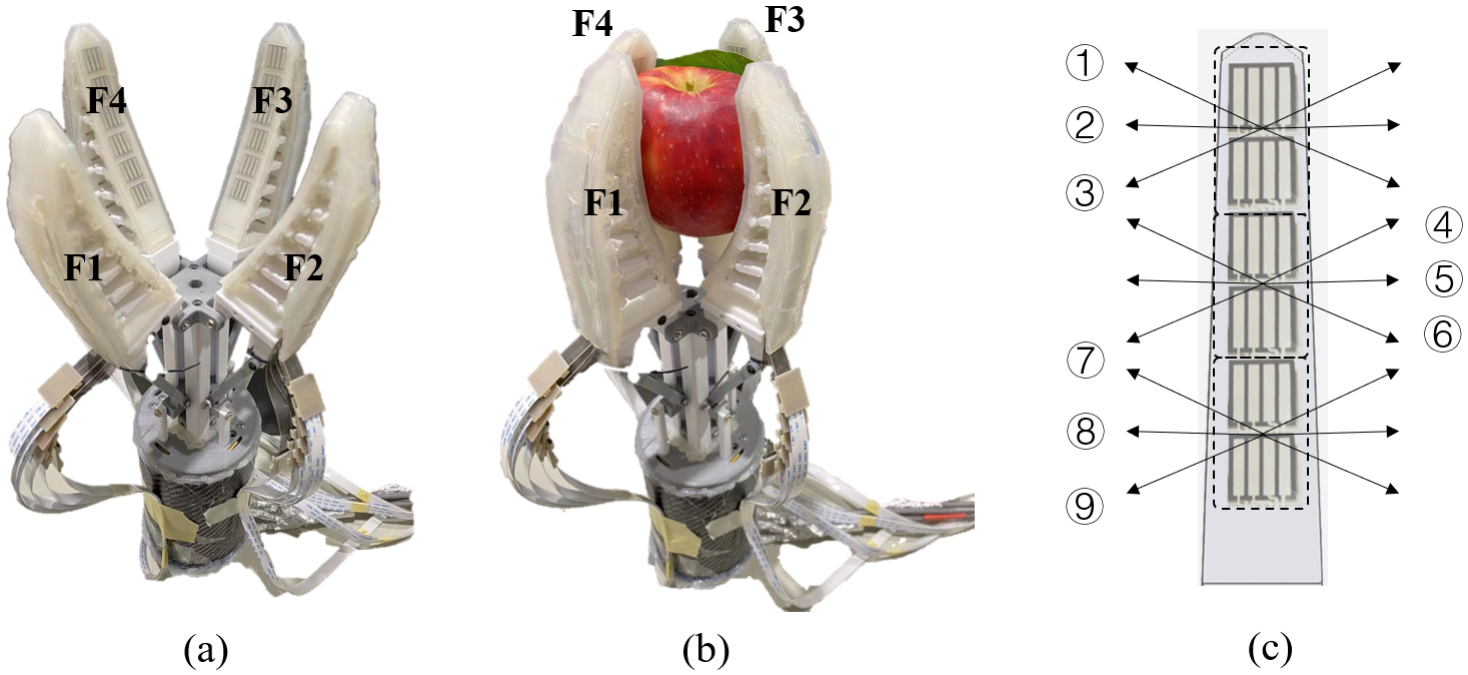
Data collection. (**a**) Gripper open; (**b**) Gripper close; (**c**) Leaf sliding directions.

To collect sufficient data for the proposed LSTM network training and validation, the sensor-integrated gripper was fixed on a table in the lab as shown in Figure 7a. We marked the four fingers as F1, F2, F3, and F4 for clear data storing and labelling. Three apples with different varieties and sizes (Pink lady No.1: dia 85 mm, height 78 mm, weight 267 g; Pink Lady No.2: dia 76 mm, height 78 mm, weight 179 g; Fuji apple: dia 72 mm, height 65 mm, weight 182 g) were prepared for grasping. For every grasp, an apple leaf with a 75 mm length and 40 mm width was placed in between one of the gripper fingers and the target apple. Once the apple and the leaf were stably grasped (as shown in Figure 7b), the leaf was manually pulled to and fro in nine different directions (① to ⑨) illustrated in Figure 7c. To make the data labelling task easier, every leaf pulling action was precisely timed so that the starting and end time frame for both non-slip and slip frames could remain the same in each data collection session. Specifically, among the 288 frames of data in one session, the 28–45 frame and the 270–288 frame were assigned for fast slips in opposite directions, while the 73–144 frame and the 172–243 frame were assigned for slow slip. The data collection sessions were repeated three times for each finger with three different apples, and the total data collected in this work included 31,104 frames.

It is important to note that the above leaf pulling motion is selected in this data collection process based on the observation and analysis of 54 leaf interfered apple grasps. A typical leaf interfered apple grasp can be divided into three stages. Specifically, in stage 1, the apple and the leaf are grasped (constrained) by the gripper fingers, and thus are moving in the same direction; no slip occurred in this stage; in stage 2, the finger interfered by leaf starts sliding together with the leaf, while the other fingers are still moving together with the apple. In this stage, the leaf, apple, and fingers are still moving in the same direction, but due to larger resistance between finger and apple, the fingers not affected by leaf interference are moving slower than leaf-interfered finger(s), which can be regarded as relatively moving to the opposite direction. In stage 3, the leaf encounters pulling force exerted by the stem, which will lead to either breaking of the leaf or pulling the leaf out of the gripper. In this stage, the leaf is moving in the opposite direction of both the finger and the apple. Therefore, this leaf-pulling process in the lab can be used to simulate the motion stage 2 and stage 3 of the leaf-interfered grasp. Another point worth mentioning is that the leaf pulling force/speed is unlikely to cause issues for robot operation. According to Decoteau [54], Craker [55], and Tong’s research [56], the 0.3–3.0 N break force required to break a leaf stem is much less than the required pulling force (up to 57.6 N) to detach an apple, not to mention that the tensile strength of leaf tissue is even smaller than the leaf stem. Considering that the commonly used Universal Robot 5 (UR5) robot can withstand forces greater than 100 N, the influence of the leaf-pulling force on the robot operation can be ignored. Similarly, the leaf-pulling speed that can be achieved by the UR5 robot is between 0–157 mm/s (calculated by transforming the maximum joint rotational speed 180${}^{\circ }$/s into linear speed on the surface of an apple with 100 mm diameter), while the limit speed of the UR5 robot is larger than 500 mm/s; therefore, the speed of the leaf-pulling motion can hardly affect the robot operation.

### 4.3. Data Pre-Processing, Labelling and Training

For every frame, a 24 × 16 matrix was created to collect the data provided by the 384 taxels embedded in the four fingers, and the interval between two frames was set to be 60 ms. Before feeding to the proposed network, the original reading from the taxels will be normalised from 1000–4096 to 0–1, after which the 24 × 16 × t matrix will be reshaped to a 384 × t matrix (A).

As aforementioned, all the slippage events have the same starting time and ending time, and data labelling was conducted autonomously via a program to distribute stored data files. The non-slip frames were labelled as 0, and the slip frames were labelled with 1, 2, 3, and 4, respectively, to indicate which finger the slippage occurred on. After labelling, all the time series data were compressed together with a time step of 12, which means every 12 frames were packed as an input for the proposed LSTM network. Considering that the LSTM network is expected to give a prediction at the end of the 12 frames input pack, each pack was labelled the same as the last frame in the pack. To clarify, pack 1 includes frame 1 to frame 12, with a label identical to the label of the 12 th frame; pack 2 consists of frame 2 to frame 13, with a label identical to the label of the frame 13.

According to the above arrangements, the total number of slip frames was slightly smaller than the non-slip frames. To balance the data to be trained, Stratified Train-Test Splits [57] were adopted to ensure that the proportions for the five (0, 1, 2, 3, and 4) data sets were identical in both the training and the testing datasets.

The total datasets were divided into three subsets: training, validation, and test sets at a ratio of 70%:20%:10%. Test sets were randomly selected from the original datasets.

To handle the possible under-defying issues for LSTM networks, a cosine annealing method was applied to control the learning rate within the range of 0.001 to 0.00001 during the training process. The learning rate setting function is as below:

(7) $$l_{r}=lr_{min}+\frac{1}{2}\left(1+\cos\frac{\pi t}{T}\right)\left(lr_{init}-lr_{min}\right)$$

where $l_{r}$ is the learning rate; $lr_{min}=1\times 10^{-5})$, $lr_{init}$ was set as 0 at the beginning of the training, and then obtained from the Adam optimizer [58], where the cap of learning rate was set to be $1\times 10^{-3})$, *t* equals with the epoch time, T=5.

The cross entropy loss function [59] was adopted in the training process to adjust model weights. With the gradient descent method [60] implemented to minimise the loss function, a higher learning rate can help the network skip the local minimum point, while a small learning rate can help the network keep updating to achieve the global minimum. The early stopping method was applied to cope with the potential over-fitting issues.

### 4.4. Slip Detection Result

The proposed three LSTM layers network demonstrated a 94% overall slip detection accuracy, and the detailed F1-score on classifying all five data groups is listed in Table 1.

**Figure 8:**
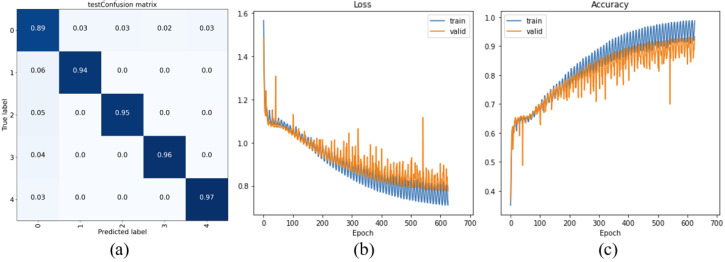
Performance of the proposed LSTM network.(**a**) Confusion matrix; (**b**) Epoch-Loss graph; (**c**) Epoch-Accuracy graph.

Figure 8 shows the confusion matrix and performance charts of the proposed LSTM network, from which we can see the model converge within less than 700 epochs. The training accuracy is above 94%.

**Figure 9:**
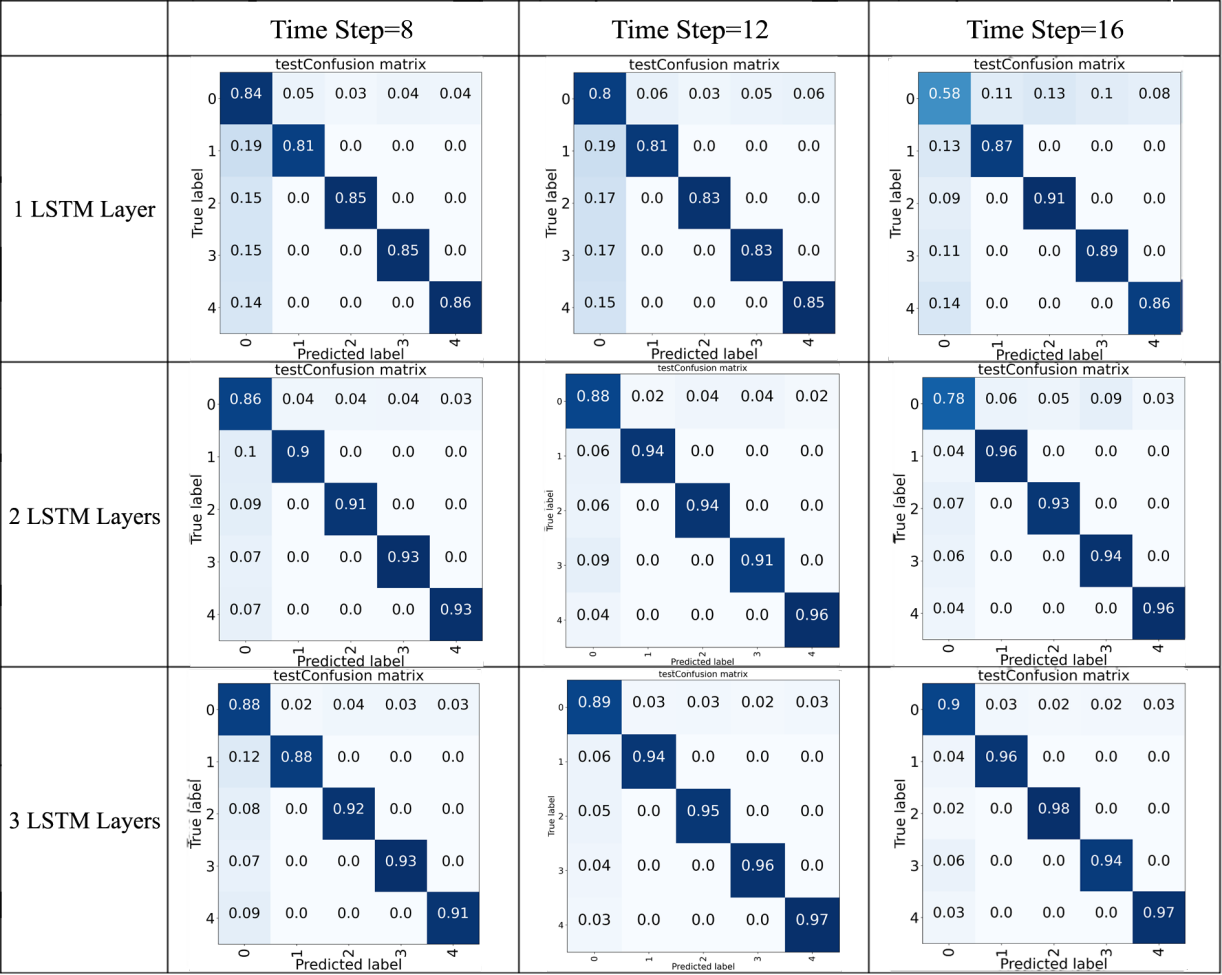
Confusion matrices for the proposed LSTM networks with different layers and time steps. (darker color represents higher accuracy.)

To examine the effect of different layer setting(s) and time step setting(s), nine different networks were tested and compared. Using the same hardware (CPU i7-7700HQ), the performance comparison between the proposed LSTM networks with different layers and time steps is presented in both Table 2 and Figure 9—from which we can see that multiple LSTM layers can significantly enhance the detect accuracy of the network model. Furthermore, in the models with two LSTM layers, networks using 12 time steps demonstrated higher average accuracy than those taking 8 time steps or 16 time steps. It is also important to note that both floating point operations (FLOPs) and the number of parameters increased with the increment of the included LSTM layers, which reflects that the computational complexity of the model with three LSTM layers is higher than the models with less LSTM layers.

However, according to our test, the computation time of the model with three layers is between 27 ms to 60 ms, which is fairly acceptable in the harvesting applications. Considering this model achieved the highest accuracy among the nine tested models, we would recommend the three layers network with either 12 time steps or 16 time steps.

### 4.5. Experiment on Grasp Manipulation

To validate the close-loop control of the proposed grasp manipulation, experiments were conducted in the Fanhauser Apples orchard in Victoria, Australia.

**Figure 10:**
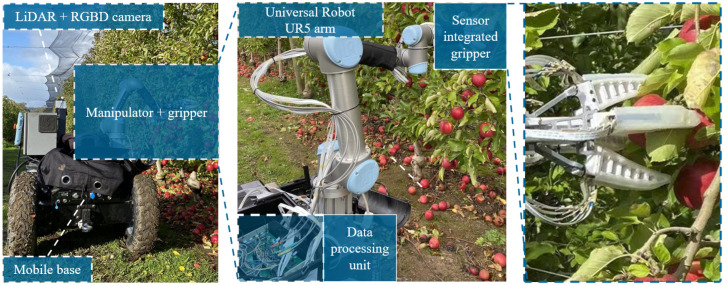
Experiment setting of grasp manipulation.

Figure 10 presents the experiment setting, where a UR5 arm was used and fixed on a mobile base together with the customised DPU. The sensor-integrated gripper was mounted on the end joint of the UR5 arm to perform the apple grasping task, and an Intel Realsense D455 camera and a DJI-Livox Mid-70 LiDAR were also mounted on the mobile base to enable machine vision for the robot system. Specifically, the color images collected by the D455 camera will be processed with 2D fruit recognition and segmentation, after which the processed data will be calibrated and fused with point cloud generated by the Mid-70 LiDAR to provide an accurate fruit location to the robot system.

**Figure 11:**
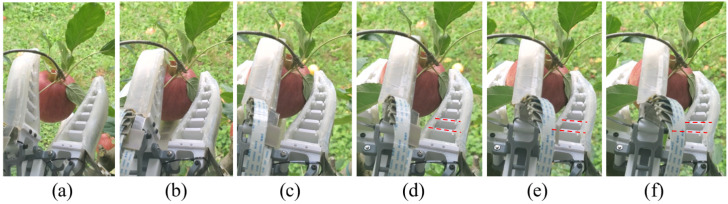
Slip detection and reaction motion sequence. (**a**) gripper arrived; (**b**) gripper closed; (**c**) gripper rotated; (**d**) slip detected; (**e**) motor-actuated finger reaction; (**f**) maximum grasping force applied.

Through 10 test(s) conducted in the field against the Pink Lady apples, the proposed LSTM network was able to detect all the noticeable slip events, and the designed grasp manipulation control proved its effectiveness. Once a slip was identified and localised on one specific finger, the corresponding servo motor was triggered to drive a timely reaction. The complete finger motion sequence can be found in Figure 11. Two red dotted lines were marked in Figure 11d–f.

To present a reference to observe the finger motion. Specifically, these two lines represent the bottom two crossbeams connecting the finger front and back. However, it can be seen that the angle between the two red dotted lines in Figure 11f is significantly smaller than those in Figure 11d, which indicates that the finger was pushed harder by the motor against the grasped apple, as planned in the grasp manipulation control loop.

### 4.6. Discussion

The proposed three-layer LSTM network achieved promising slip detection accuracy in this work. The proposed grasp manipulation approach also demonstrated its effectiveness in responding to leaf interference. It is important to note that the training dataset collected in the lab should be enlarged with extensive field test data against different fruit variety, leaf property, and environmental variables to secure its performance in various scenarios. In addition, in this work, the proposed method was applied to detect and handle slippage on a single finger. It is expected that the proposed method will be optimised in the future to cover the scenarios of leaf slip interference on multiple fingers, as well as other complex scenarios including leaf-branch combined slip interference. It is also expected that the proposed method can be extended to other pick-place applications.

## 5. Conclusions

This work proposed a learning-based method for slip detection in the fruit harvesting field. The contributions of this work are: (1) The connection between leaf interference and inadequate harvest performance has been analysed and identified, which is the first time of its kind; (2) A three-layer LSTM network has been developed and achieved a 94% slip detection accuracy, based on a set of low-cost tactile sensors; (3) The proposed slip-detection based grasp manipulation demonstrated its effectiveness in the orchard, which indicates great potential for improving robotic harvesting performance in the real world.

## Figures and Tables

**Table 1:** F1-score of the proposed LSTM algorithm.

	Precision	Recall	F1-Score
No slip	0.92	0.89	0.91
Slip on finger 1	0.94	0.94	0.94
Slip on finger 2	0.94	0.95	0.94
Slip on finger 3	0.95	0.96	0.96
Slip on finger 4	0.94	0.97	0.96
Accuracy	-	-	0.93
Macro avg	0.94	0.94	0.94
Weighted avg	0.93	0.93	0.93

**Table 2:** Performance comparison of the proposed LSTM networks.

	Best Accuracy	Average Accuracy ± Stdev	Model Parameters	FLOPs
LSTM1-8	89%	86% ± 0.04	26,373	1.41k
LSTM1-12	88%	84% ± 0.05	26,373	1.41k
LSTM1-16	85%	80% ± 0.09	26,373	1.41k
LSTM2-8	93%	91% ± 0.03	34,373	4.83k
LSTM2-12	96%	93% ± 0.03	34,373	4.83k
LSTM2-16	95%	90% ± 0.05	34,373	4.83k
LSTM3-8	93%	91% ± 0.03	65,733	17.82k
LSTM3-12	96%	94% ± 0.02	65,733	17.82k
LSTM3-16	97%	95% ± 0.02	65,733	17.82k

## Abbreviations

The following abbreviations are used in this manuscript:

LSTM	Long-short term memory
COM	Center of mass
DPU	Data processing unit
DOF	Degree of freedom
TPU	Thermoplastic polyurethane
FFC	Flat flex cables
RNN	Recurrent neural network
CNN	Convolutional neural network
FLOPs	Floating point operations
